# Supplementary material for: Comparative Transcriptome Analysis of the Molecular Mechanism of the Hairy Roots of Brassica campestris L. in Response to Cadmium Stress
Source: Int J Mol Sci. 2019 Dec 26;21(1):180. doi: 10.3390/ijms21010180 (PMC6981690; doi:10.3390/ijms21010180)
Supplement: Supplementary file 1 [file ijms-21-00180-s001.zip › Supplementary/Supplementary figure.docx]

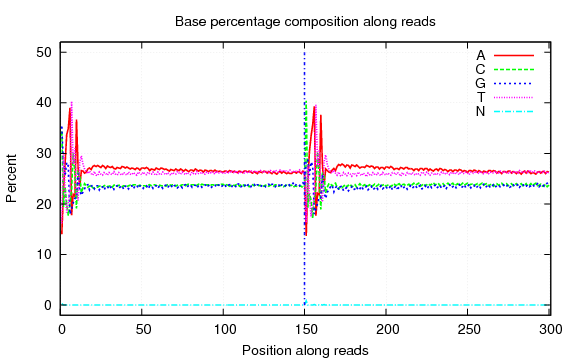


Figure.S1. Distribution of base composition on clean reads. X axis represents base position along reads. Y axis represents base content percentage.


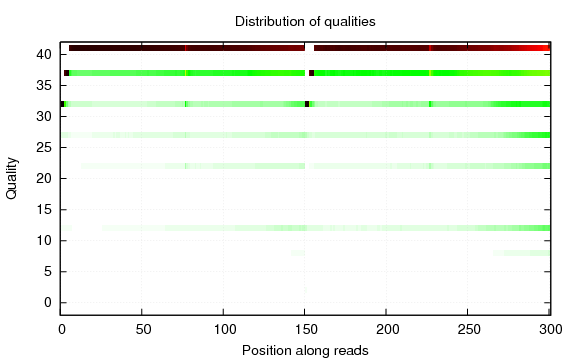


Figure.S2. Distribution of base quality on clean reads. X axis represents base positions along reads. Y axis represents base quality value.


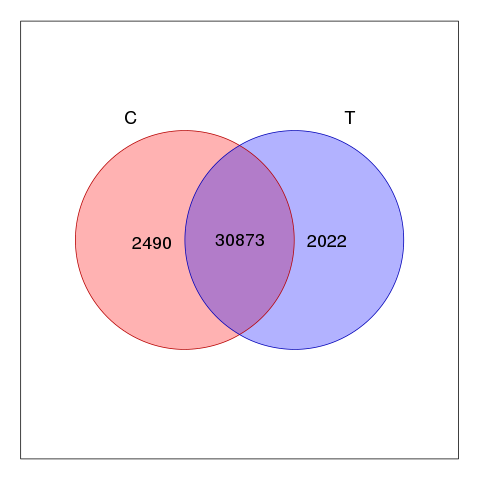


 Figure.S3. Venn diagram analysis


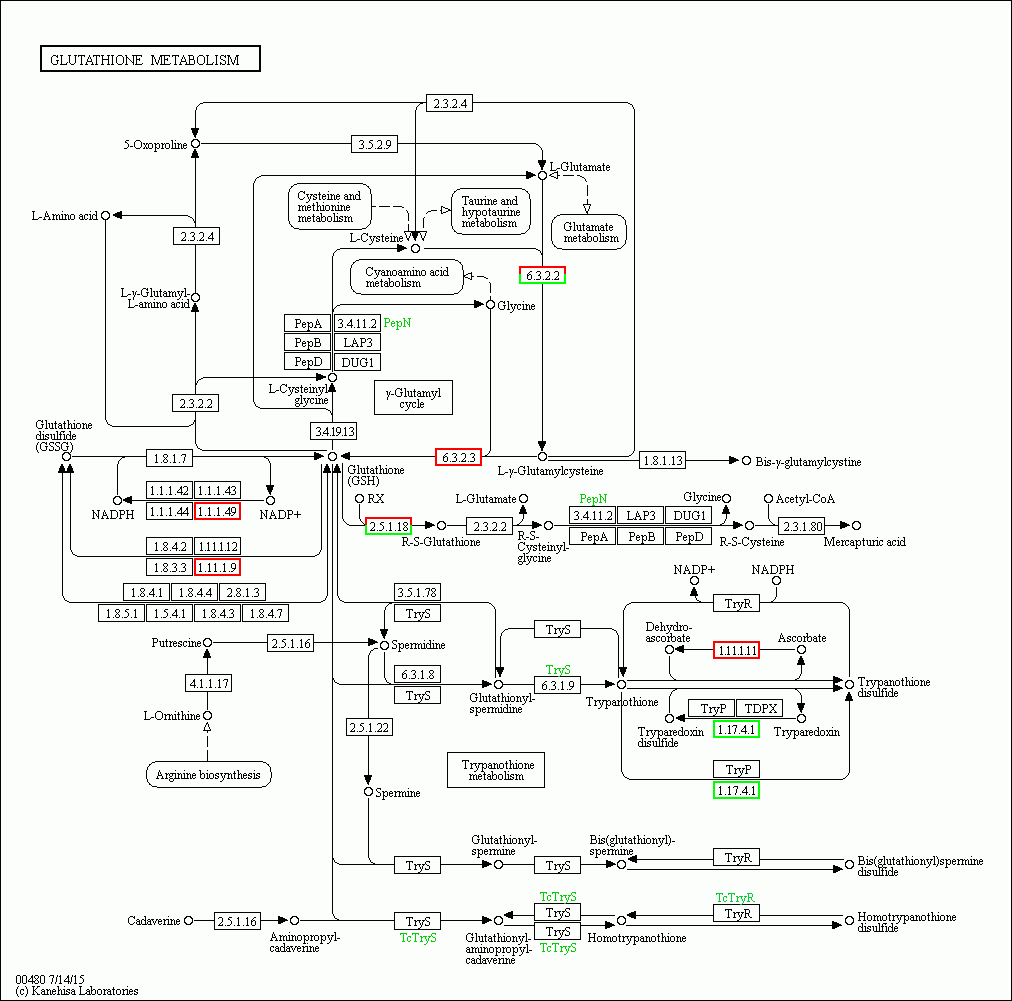


**Figure S4.** Glutathione metabolism in *Brasssica campestris L.*

**Figure S5.** Molecular Function with GST unigene
